# Blood Glutamate Scavenging: Insight into Neuroprotection

**DOI:** 10.3390/ijms130810041

**Published:** 2012-08-13

**Authors:** Akiva Leibowitz, Matthew Boyko, Yoram Shapira, Alexander Zlotnik

**Affiliations:** Department of Anesthesiology and Critical Care, Soroka Medical Center, Ben-Gurion University, Beer Sheva 84894, Israel; E-Mails: mathewb@bgu.ac.il (M.B.); yoramsh@clalit.org.il (Y.S.); zlotnika@bgu.ac.il (A.Z.)

**Keywords:** glutamate, scavenging, neuroprotection, brain ischemia, traumatic brain injury

## Abstract

Brain insults are characterized by a multitude of complex processes, of which glutamate release plays a major role. Deleterious excess of glutamate in the brain’s extracellular fluids stimulates glutamate receptors, which in turn lead to cell swelling, apoptosis, and neuronal death. These exacerbate neurological outcome. Approaches aimed at antagonizing the astrocytic and glial glutamate receptors have failed to demonstrate clinical benefit. Alternatively, eliminating excess glutamate from brain interstitial fluids by making use of the naturally occurring brain-to-blood glutamate efflux has been shown to be effective in various animal studies. This is facilitated by gradient driven transport across brain capillary endothelial glutamate transporters. Blood glutamate scavengers enhance this naturally occurring mechanism by reducing the blood glutamate concentration, thus increasing the rate at which excess glutamate is cleared. Blood glutamate scavenging is achieved by several mechanisms including: catalyzation of the enzymatic process involved in glutamate metabolism, redistribution of glutamate into tissue, and acute stress response. Regardless of the mechanism involved, decreased blood glutamate concentration is associated with improved neurological outcome. This review focuses on the physiological, mechanistic and clinical roles of blood glutamate scavenging, particularly in the context of acute and chronic CNS injury. We discuss the details of brain-to-blood glutamate efflux, auto-regulation mechanisms of blood glutamate, natural and exogenous blood glutamate scavenging systems, and redistribution of glutamate. We then propose different applied methodologies to reduce blood and brain glutamate concentrations and discuss the neuroprotective role of blood glutamate scavenging.

## 1. Introduction

Glutamate, a known excitatory neurotransmitter in the central nervous system (CNS), is a non-essential amino acid, and is the most abundant free amino acid in the CNS, accounting for approximately 60 percent of total neurotransmitter activity in the brain. Glutamate has several important roles in the development and function of normal brain activities, amongst them: regulation of the communication process between neurons, development of plasticity in the CNS, and serving as an energy reserve [1–5]. Activation of *N*-Methyl d-Aspartate (NMDA) receptors by glutamate is vital for brain function. They are central to many of the activity-dependent changes in synaptic strength and connectivity that are thought to underlie the formation of memory and learning. There is growing evidence that physiological levels of synaptic NMDA receptor activation promote survival of many types of neurons or render them more resistant to trauma [6]. The beneficial effects of glutamate are greatly dependent on strict homeostasis, maintaining brain extracellular fluid (ECF) glutamate at concentrations below their toxic range. The low concentration (0.3–2 μM/L) [1] of glutamate in the brain’s ECF is maintained by a well-established mechanism of compartmentalization of glutamate. Glutamate released from neurons as a neurotransmitter stimulates glutaminergic receptors (ionotropic NMDA and AMPA receptors or metabotropic glutamate receptors). Excessive activation of ionotropic receptors leads to the opening of receptor-coupled ionophores, including calcium channels, which are of particular importance. Efflux of calcium into neurons, which activates plasmatic proteolytic enzymes, results in neuronal death via apoptosis or necrosis [7–11]. Animal models and human clinical studies reveal the association of pathologically elevated ECF glutamate levels and several acute and chronic neurodegenerative disorders, including stroke [12], traumatic brain injury (TBI) [13], intracerebral hemorrhage [14], meningitis, brain hypoxia [15], amyotrophic lateral sclerosis (ALS) [16], glaucoma [17], HIV dementia [18], glioma [19] and many others. These states are characterized by a several hundred-fold elevation of glutamate concentration in the brain’s ECF facilitated by blood brain barrier (BBB) breakdown, permitting free movement of glutamate between plasma and ECF, along its concentration gradient.

Mechanisms counteracting glutamate-induced excitotoxicity include a large family of Na^+^ dependent glutamate transporters known as excitatory amino acid transporters (EAATs). They are localized on cell membranes of neurons, astrocytes and the endothelium of the BBB [1,8]. Glutamate released as a transmitter at the synapse is taken up by surrounding astrocytes and amidated to glutamine, a non-neuroexcitatory amino acid, which is then transferred back to neurons to be converted back to glutamate [20,21].

Alternative mechanisms exist as well. Glutamate has been found to move unidirectionaly from brain into blood, a process facilitated by the presence of glutamate transporters on the abluminal membrane of brain endothelial cells. These glutamate transporters include: EAAT1, EAAT2 and EAAT3, enabling high affinity, sodium-dependent glutamate transport into the endothelial cells [22–25]. They also harbor glutamine transporters that take up glutamine at the abluminal side of the membrane. In endothelial cells, glutamine is transformed into glutamate via glutaminase, resulting in glutamate build up, eventually reaching an endothelial cell concentration which is much higher than that of plasma. Glutamate is then transported into blood via facilitated diffusion, down an electrochemical gradient, reaching an average blood plasma concentration of 40 μM/L. This mechanism principally allows the efflux of glutamate from brain-to-blood despite the very unfavorable brain-to-blood glutamate concentration gradient. A low capacity glutamate carrier exists on the luminal membrane of endothelial cells. The transport capacity for influx of glutamate from blood into brain is quite low, reaching 80% saturation at normal plasma concentrations. This results in a non-significant rate of glutamate influx into the brain [26–28].

Despite their role in brain glutamate regulation, little attention has been given to the glutamate transporters present on brain blood vessels [25,29]. Their role in controlling brain extracellular glutamate has been generally ignored as an important glutamate removal mechanism [30]. Recently, the brain-to-blood glutamate efflux has attracted renewed interest. In rats, glutamate injected into cerebrospinal fluid is removed into the blood within a relatively short time period. Eighty percent of radio-labeled glutamate injected into the cisterna magna was detected in rats’ peripheral blood within a few minutes after injection [31]. This demonstrates the capacity of glutamate transporters on the choroid epithelium forming the CSF-blood barrier to remove excess glutamate. These glutamate transporters, which are involved in removing the excess glutamate, are thought to exist both on the vascular endothelium forming the blood–brain barrier within the brain interstitial compartment, and on the choroid epithelium forming the blood–cerebrospinal fluid (CSF) barrier [24,28]. Other studies, investigating the removal of glutamate from brain parenchyma to blood through the ISF-blood barrier confirm this finding [32]. Removal of glutamate from brain ECF into blood is attributed to the existence of the abluminal glutamate transporters, and the highly vascularized nature of the brain, which is comprised of an extensive network of capillary blood vessels, with an average capillary-to-capillary distance of 19 ± 4 μm, a total capillary surface of 12 m^2^, and an average capillary distance of 8–20 μm. This vascular network accounts for the proportionally high cerebral blood (reaching 20% of total cardiac output), which allows this mechanism to be effective [33–35].

Recent research applies different strategies for minimizing the toxic effects of glutamate in the context of ischemic stroke and traumatic brain injury (TBI). This provides neuroprotection, including: inhibiting glutamate synthesis, blocking its release from presynaptic terminals, antagonizing its actions on postsynaptic receptors, and accelerating its reuptake from the synaptic cleft. While animal models of ischemia [36] have provided evidence supporting the role of NMDA receptor antagonists in neuroprotection, clinical trials using NMDA receptor antagonists following stroke and TBI have failed to provide significant neurological improvement. In some instances they have been shown to be harmful, deteriorating neurological outcomes and increasing the mortality rate [36–39]. NMDA receptor antagonists, which do not discriminate between the different actions of the receptor interfere with both the negative and positive effects of this signaling [6,39]. Their effect extends beyond the brain: They antagonize extra-cerebral glutamate transporters such as those in the pancreas [40–44] and affect the metabolic regulation of glutamate.

Alternative treatment modalities that focus on elimination of the excess toxic glutamate, rather than antagonizing the receptors have been developed. Teichberg *et al.*, postulated that excess of glutamate in the brain’s ECF may be safely and effectively removed into the plasma via the endothelial transporter systems [45,46]. Under normal circumstances, plasma glutamate concentration is 5–100 μM/L [1], the whole blood concentration is 150–300 μM/L [47,48] while in the brain’s ECF it is only 0.3–2 μM/L. Removal of excess glutamate from the brain to the blood involves overcoming significant concentration gradients. Decreasing the plasma/blood glutamate concentration reduces this unfavorable gradient, and facilitates efflux of glutamate from the brain into the blood, which is termed “neuroprotection by blood glutamate scavenging”. Compounds capable of reducing blood glutamate levels are designated “blood glutamate scavengers”. The activity of blood glutamate scavengers in stimulating brain-to-blood glutamate efflux is self-limiting, since the process slows down and stops when the excess glutamate levels have decreased to concentrations below the threshold of activation of the brain vasculature glutamate transporters (*i.e.*, below their Km values) which mediates the glutamate efflux into the blood. Thus, blood glutamate scavengers should preserve the physiological effects of glutamate in regulating the metabolic and electrolyte balance, maintaining neuronal function. Supplementary Figure S1 graphically summarizes the homeostasis of glutamate in brain extracellular fluids.

## 2. Relationship between Blood/Plasma and ECF Glutamate Levels

Berl *et al.*, [30] were the first to show the existence of a brain-to-blood glutamate efflux following intracisternal administration of [^14^C]-glutamate. Within 1 min, 25% of the injected [^14^C]-glutamate was found in the plasma. This was later confirmed by Davson *et al.*, [49] and by Al-Sarraf *et al.*, [50] who demonstrated a concomitant decrease in CSF glutamate and rise in plasma, following ventriculo-cisternal administration of radio-labeled glutamate. Gottlieb *et al*. reproduced this effect, showing an immediate appearance in blood and enhanced disappearance from the CSF, following a decrease of blood glutamate levels [31]. Other studies, demonstrated clearance of glutamate from brain parenchyma to blood through the ISF-blood barrier as well [32]. This brain-to-blood glutamate efflux was shown to be accelerated by expanding the glutamate concentration gradient between the cerebrospinal fluid/capillary endothelial cell and the blood plasma. These results were later confirmed using dual-probe brain microdialysis demonstrating accelerated elimination of excess glutamate from the brain parenchyma following intravenous administration of blood glutamate scavengers, and decelerated elimination following intravenous administration of glutamate [46]. Using functional magnetic resonance imaging (MRI) in a rat model of stroke, Campos and colleagues showed that decreasing plasma glutamate levels with blood glutamate scavengers was associated with a significant decrease of glutamate in the brain [51] correlated with neurological improvement. Abu Fanne and colleagues reported that the reduction of glutamate concentration in plasma was associated with a decrease of glutamate in CSF after middle cerebral artery occlusion (MCAO) in rats [52] and after TBI in mice [53]. Clinical studies in healthy volunteers and neurosurgical patients showed a close correlation between plasma glutamate concentrations and CSF concentrations [54,55]. Similar results were seen in different neurodegenerative disorders including stroke, HIV dementia [18,56], and migraine [17]. In human stroke, Castillo *et al.*, showed that lower plasma glutamate levels correlated with lower concentrations of glutamate in patients’ cerebrospinal fluid (CSF) [12,57]. Some studies seem to question this correlation [58]. These discrepancies may be at least partially resolved by the distinct methodology used in the studies (functional MRI and collection of plasma samples within a one week interval).

## 3. Effect of Blood Glutamate Levels on Neurological Outcome and Their Association with Different Neurodegenerative Disorders

Extensive data reveals a relationship between blood glutamate levels and different neurodegenerative conditions, possibly reflecting the role of blood glutamate as an etiological factor rather than a result of disease.

### 3.1. TBI (Traumatic Brain Injury)

Blood glutamate levels have been shown to inversely correlate with neurological outcomes in rat models of traumatic brain injuriy (TBI), while administration of scavengers-oxaloacetate, pyruvate, GOT and GPT has been shown to significantly improve neurological outcomes [46,59–63]. Conversely, artificial elevation of blood glutamate concentration deteriorated neurological outcome [59,61]. An ongoing brain-to-blood glutamate efflux in comatose TBI patients has been indicated by an increase in arterio-venous jugular glutamate differentials [64].

### 3.2. Stroke

In rat models of permanent middle cerebral artery occlusion (MCAO), the neurological outcome inversely correlated with blood [65] or plasma [51] glutamate levels. A three-fold increase of plasma glutamate compared with baseline values has been shown to begin 4–6 h after ischemic induction, reaching peak values at 8–24 h and returning to pre-ischemic values by 48–72 h [66]. Human plasma glutamate levels during the first 24 h of stroke were shown to correlate with the volume of ischemic lesion on CT scans or MRI and neurological outcomes [12,57,67,68].

### 3.3. Chronic Renal Failure (CRF)

Plasma and whole blood glutamate levels have been shown to be significantly elevated in patients with chronic renal failure (CRF) [69–71]. Insulin like growth factor binding protein (IGFBP) is elevated in patients with end stage renal failure, resulting in reduced bioactivity of insulin like growth factor (IGF-1) [72]. IGF-1 has been shown to significantly decrease blood glutamate levels. This effect is more pronounced in healthy patients compared with patients suffering from chronic renal failure (CRF) [72]. Elevated levels of IGFBP promote elevation of plasma glutamate levels. CRF, which is known to be associated with elevated levels of IGFBP, results in very high concentrations of glutamate in both plasma and whole blood of the patients with CRF. In conditions involving wasting of muscle mass, muscle glutamate has been shown to be the only amino acid to inversely correlate with RBC glutamate. Blood glutamate remains elevated in patients with CRF and has been shown to decrease following hemodialysis, correlating with a decrease in urea [73]. Thus, elevated glutamate levels found in blood of patients with CRF and ARF may contribute to uremic encephalopathy [74,75].

### 3.4. Depression

Several studies associate major depression with elevated blood glutamate levels compared with healthy controls [76–79]. Antidepressants promoted decrease of blood glutamate levels, though they did not descend to normal values [77].

### 3.5. Dementia

Patients suffering from HIV dementia were shown to have elevated plasma glutamate levels, which correlated with the clinical severity of dementia [18,56]. Antiviral treatment with azydodeoxythymidine leads to a decrease of plasma glutamate levels compared with non-treated patients [80].

### 3.6. Schizophrenia

Data available is inconsistent. While some publications demonstrate similar plasma glutamate levels in schizophrenics and controls, others report that plasma glutamate levels are significantly higher in schizophrenic patients, particularly in those resistant to neuroleptics [81,82].

### 3.7. Epilepsy

In the single study performed, no difference in plasma glutamate levels was found between patients and controls. However, responders to the antiepileptic drug Lamotrigine had significantly lower plasma glutamate levels compared with non-responders [83].

### 3.8. Amyotrophic Lateral Sclerosis (ALS)

Some studies find significant elevation of plasma glutamate levels in patients with amyotrophic lateral sclerosis (ALS), which correlated with the duration but not severity of the disease. Others demonstrated this correlation only in spinal but not bulbar forms of ALS [16,84–86].

### 3.9. Other Disease States

Plasma glutamate has been shown to be elevated in patients with rheumatoid arthritis with temporal-mandibular joint crepitus [87,88]. Levels of glutamate in plasma were significantly elevated in patients with motor neuron disease [89], in patients with alcoholic liver disease [90], and Parkinson’s disease [86].

## 4. The Contribution of Blood Glutamate Reduction to Neuroprotection

Autoregulatory mechanisms stabilize plasma glutamate levels in different circumstances, maintaining glutamate in a relatively narrow range. Several complex and poly-component mechanisms participate in this process.

### 4.1. Oxaloacetate, Pyruvate, GOT and GPT

Liver delivered plasma resident enzymes glutamate-oxaloacetate transaminase (GOT) and glutamate-pyruvate transaminase (GPT) in the presence of their co-substrates, oxaloacetate and pyruvate, convert glutamate into 2-ketoglutarate, aspartate and alanine [31]. Gottlieb *et al.*, showed *in vivo* that 1 mM pyruvate injected intravenously was capable of decreasing blood glutamate levels by 30%. In addition, 1 mM oxaloacetate injected intravenously decreased glutamate in blood by 40%, and the combination of the two decreased glutamate as much as by 60% below baseline levels [31]. Subsequently, blood glutamate rose with a decrease in CSF glutamate, reflecting the movement from CSF to blood. This capacity was demonstrated *in vitro* as well, and has been repeated in various pathological states such as TBI and stroke [51,52,59–61,65], and correlated closely with neurological outcome. Interestingly, blood glutamate scavenging activity has been shown to be dose-related. When injected intravenously as a continuous infusion of 30 min duration starting 60 min after TBI or stroke, oxaloacetate had a maximal effect in 1 M solutions, with a gradually decreasing effect with lower doses until the effect disappeared at a dose of 0.01 M [60]. Pyruvate showed a similar dose-response pattern [61]. In a model of stroke, the neuroprotective effect was maximal at 0.25 M, decreasing thereafter [65]. Previous studies have also suggested a ∩-shape dose-response curve for pyruvate, where pyruvate loses its efficacy at doses higher than 250 mg/kg [91]. The discrepancy between oxaloacetate and pyruvate leads to a conclusion that pyruvate-induced neuroprotection is at least partially mediated via mechanisms not related to blood glutamate scavenging activity [51,61,65]. Oxaloacetate effectively improved short- and long-term neurological outcomes after moderate to severe TBI, reduced the extent of brain edema and improved survival of neurons in different regions of the hippocampus [59,60,92]. Pyruvate demonstrated similar results both in TBI and stroke, with improved neurological outcomes, reduced mortality rate, decreased BBB permeability, smaller lesions on brain histological examination, and increased neuronal survival [60,61,92].

The neuroprotective role of oxaloacetate and pyruvate may be related to several additional mechanisms, including the activation of pyruvate dehydrogenase to restore cellular ATP levels. It acts as a direct antioxidant, reacts with H_2_O_2_ to form water and carbon dioxide, scavenges hydroxyl radicals, has the ability to stimulate NADPH-dependent peroxide scavenging systems, inhibits poly(ADP-ribose) polymerase activity, and has the capacity to serve as an alternative source of energy thereby improving the brain’s energetic and redox status [93–96]. Several rather compelling arguments support the contention that oxaloacetate and pyruvate exert their neuroprotective effects mainly via blood glutamate scavenging.

- Neurological recovery after TBI is not observed in rats treated with a combination of oxaloacetate and glutamate or pyruvate and glutamate. This finding is in line with the view that the presence of excess glutamate in blood prevents oxaloacetate or pyruvate to effectively exert its blood glutamate scavenging action, which is necessary for a therapeutic enhanced efflux of excess glutamate from brain into blood. If oxaloacetate and pyruvate were to exert their therapeutic effect in the brain itself, it is not clear why this action would be prevented by intravenous excess of glutamate, since the entrance of oxaloacetate or pyruvate first from the blood into the brain and then further into neurons takes place via dicarboxylate transporters such as the NaDC-3 which are not inhibited by glutamate [97,98]. One can further argue that the glutamate induced neutralization of the therapeutic effect of oxaloacetate or pyruvate must be exerted within the blood and not within the brain since glutamate does not significantly penetrate from blood into brain neither in normal physiological conditions [99] nor after the breakdown of the blood brain barrier [100].
- The oxaloacetate- and pyruvate-induced neurological recovery after TBI takes place following the administration of oxaloacetate or pyruvate within the time frame of elevated glutamate in the brain, *i.e.*, up to 2 h post TBI [101–106]. If the actions of oxaloacetate or pyruvate were unrelated to blood glutamate scavenging but rather to an improvement of mitochondrial energetics, the observation that the therapeutic effect of oxaloacetate and pyruvate is limited to two hours post TBI cannot be easily reconciled with the observation that mitochondrial dysfunction is observed for several hours after TBI [107–109].
- Neurological recovery after TBI and stroke has been shown to be dependent on two related determinants: oxaloacetate + GOT and pyruvate + GPT, while the actions of oxaloacetate and pyruvate were found to be dose dependent. Neurological recovery of rats subjected to TBI and treated IV with 5 nmol/100 g pyruvate (*i.e.*, at a dose which by itself has no detectable therapeutic effect and produces no decrease of blood glutamate levels) is significantly increased by the of 60 μg GPT/100 g rat, suggesting the involvement of blood GPT and of its blood glutamate scavenging activity in the neurological recovery. The fact that GPT alone causes a decrease in blood glutamate levels and is neuroprotective can be attributed to the fact that the endogenous blood pyruvate concentration is about 0.15 mM and close to the pyruvate/GPT Km value [61,65]. Studies with oxaloacetate reached similar conclusions. Sub-therapeutic doses of oxaloacetate did not exert any scavenging or neuroprotective activity, while the addition of 0.14 nmol recombinant GOT/100 g rat completely restored the glutamate scavenging and neuroprotective activity of oxaloacetate [59]. The tight therapeutic synergism between blood oxaloacetate and GOT as well as pyruvate and GPT is also difficult to reconcile with the suggestion of an intraparenchymal site of action, as both oxaloacetate/GOT and pyruvate/GPT would need to be translocated from blood into brain to exert their beneficial effects. The respective kinetics of intraparenchymal entry of oxaloacetate (which is specifically transported via a dicarboxylate transporter) and GOT (which cannot gain access into the brain via an intact BBB) are probably incompatible with a synergy of action since the BBB opening that begins at the time of the trauma is transient, and lasts no more than 30 min [110], while GOT is administered after 60–90 min post trauma.
- Administration of oxaloacetate in the presence of maleate, a GOT inhibitor, abolished the oxaloacetate-induced improvement in the neurological severity score (NSS, a motor–behavioral score) as well as the oxaloacetate-induced decrease of blood glutamate concentration. If the neuroprotective effect of oxaloacetate would be related to any mechanism different from blood glutamate scavenging, blockade of GOT with maleate could not prevent neuroprotection by oxaloacetate [60].

In contrast to rat models of stroke and TBI, treatment of humans with oxaloacetate potentially has serious limitations. The required dosage of oxaloacetate in humans, corresponding to that used in rats, may be toxic. The average human has 5 liters of blood, and the 1 mL solution that is likely to be injected into stroke patients should contain, as in rats, about 1 mmol of oxaloacetate. As this solution ought to be at a neutral pH, about 2 mmol NaOH are added to neutralize the acidity of oxaloacetate. Thus, the injected solution should be 10^−3^ mmol. This would require 5 M/L of Oxaloacetate and 10 M/L NaCl, which will obviously not be tolerated by the patient. Nevertheless, following the Michaelis-Menten enzyme rate equation supplementing the solution with GOT may relieve the need for high oxaloacetate concentrations [111].

Treatment with intravenous GOT is unlikely to carry unwanted pathological consequences. Plasma glutamate fluctuates by 50% during the circadian cycle [112], most likely due to the accumulation of glutamate in brain fluids during intense neuronal activity or the REM phases of sleep. GOT may transiently increase several hundred-fold, such as in hepatitis, without any known sequel-transient or permanent.

GOT and GPT concentrations in rodent plasma are significantly higher than in human plasma (GOT 132 ± 36 IU/L *vs.* 24 ± 4 IU/L, GPT 59 ± 12 IU/L *vs.* 27 ± 9 IU/L) [47]. This striking difference may partially explain the excellent blood glutamate scavenging capacity and be at least partially responsible for the better and more complete neurological recovery after TBI and stroke in animal models.

### 4.2. Stress and Activation of β_2_ Adrenergic Receptors

In animal models, a spontaneous decrease of blood glutamate levels is noticed between 60 and 120 min after infliction of TBI [59–63]. Thomassen *et al.*, reported a significant decrease in plasma glutamate in the initial six hours of myocardial infarction in humans. This decrease was accompanied by a long lasting elevation of alanine, the end product of glutamate biotransformation, reflecting accelerated breakdown of glutamate [113]. This decrease is hypothesized to be part of a hypothalamo-pituitary-adrenal stress response typically seen after TBI, stroke, SAH and myocardial infarction. Adrenaline and noradrenaline blood levels increase following TBI; their circulatory levels can rise up to 500-fold and 100-fold, respectively [114], and remain elevated several days after injury [115]. Attempts were made to establish the specific components of stress response responsible for glutamate-reducing effect and neuroprotection. Pretreatment with propranolol (a non-selective β-antagonist) completely prevented the stress-induced glutamate decrease and abolished the spontaneous partial neurological improvement typically seen at 24–48 h after TBI [62,63]. Corticotrophin releasing factor (CRF) and adrenaline significantly and consistently decreased blood glutamate levels in naïve rats, while adrenocorticotrophic hormone (ACTH) and noreadrenaline had no effect [62]. Antalarmine (a selective type-1 CRF receptor antagonist) occluded the CRF-mediated decrease in blood glutamate levels. Treatment with isoproterenol (a β_1,2_-selective adrenoreceptor agonist) produced a marked sustained decrease in blood glutamate levels. These results suggest that stress induces a decrease of blood glutamate levels partly via the activation of peripheral CRF receptors and the activation of the β-adrenoreceptors [62]. Using a combination of different non-selective and selective β adrenergic agonists and antagonists we showed that activation of β_2_ receptors plays an important role in the homeostasis of glutamate, decreasing its levels in rat blood and promoting neuroprotection. Conversely, activation of β_1_ adrenergic receptors causes moderate elevation of glutamate in blood [47,62,63,116]. The precise mechanism by which β_2_ receptors cause this effect remains unclear. At least two possible mechanisms may be involved. First, redistribution of unchanged glutamate from the blood into peripheral tissues, such as skeletal muscle, gut and liver may be enhanced. Those tissues have been shown to be responsible for absorption of 90% of glutamate injected into the bloodstream within 10 min [117]. The second mechanism may be due to activation of endogenous GOT and GPT as part of the stress response, eventually leading to conversion of glutamate into 2-ketoglutarate, alanine and aspartate [118].

Stress after injury is known to activate an adaptive response, which allows the body to marshal its forces to confront a threat and provide protection. Although a late surge of catecholamines may be detrimental [119], the stress-related release of adrenaline may serve to reduce brain injury by limiting the increase of glutamate in the brain’s fluids after head injury.

### 4.3. Insulin and Glucagon

The effect of insulin and glucagon on amino acid and glucose metabolism was studied extensively. Aoki *et al.*, showed on healthy volunteers that insulin increased uptake of glutamate into muscle [120]. The authors measured glutamate concentrations separately in plasma and whole blood and showed that insulin had a very limited effect on glutamate concentration in plasma, but it significantly reduced glutamate concentration in whole blood. This effect could be explained by a concomitant shift of glutamate from red blood cells (RBC) as an attempt to maintain a stable concentration of glutamate in plasma while a 9 k plasma-to-muscle glutamate shift takes place [120]. Leweling and colleagues found that plasma glutamate levels are decreased in patients with hepatic failure and hyperammonemia, which is known to be a potent activator of insulin secretion. The authors suggested that lower glutamate levels seen in patients with hepatic failure were a sequence of hyperinsulinemia. Alanine (a metabolite of glutamate breakdown) is also decreased in those patients, suggesting that glutamate is decreased as a result of its shift into skeletal muscle or slower synthesis of glutamate [121]. IGF-1 has been shown to significantly decrease blood glutamate levels.

Glucose, insulin and glucagon have all been shown to significantly reduce blood glutamate levels [116]. The glucose induced glutamate reducing effect is probably related to the stimulating effect of glucose on insulin secretion [122]. In turn, elevated insulin promotes inflow of glutamate from plasma into skeletal muscle [123]. In contrast, elevated glutamate probably initiates secretion of both insulin and glucagon as part of a positive feedback mechanism, returning glutamate towards normal values. Previous studies have shown that glutamate stimulates both glucagon [124,125] and insulin release [123,126–128] by acting on a glutamate receptor of the AMPA subtype in the pancreas to lower glutamate back to normal levels. It has been further described that glutamate exerts a much weaker stimulation for the secretion of glucagon than it does for the secretion of insulin [124]. Despite having similar glutamate-scavenging properties, insulin and glucagon exhibit different glutamate reducing patterns. Administration of insulin led to an immediate and transient decrease in blood glutamate levels, while glucagon led to a delayed but long-lasting decrease in blood glutamate levels. Abu Fanne *et al.*, showed that both insulin and glucagon treatments effectively reduce plasma and CSF glutamate levels in rats submitted to MCAO and in this way improved neurological outcome [52]. Glucagon induced hyperglycemia did not exacerbate neurological outcome in this study, despite the controversial clinical and experimental data suggesting that hyperglycemia is associated with a worse neurological outcome (although recent data does not support this notion) [129,130]. The same group observed a significant neuroprotective effect in mice treated with glucagon prior to or after TBI [53]. This neurological improvement correlated with lower plasma and CSF glutamate concentrations. The authors postulated that the neuroprotective effects of both insulin and glucagon were due to their blood and ECF glutamate scavenging effect.

### 4.4. Estrogen and Progesterone

There is substantial biologic evidence for the importance of the role of estrogen and progesterone in the development, growth, differentiation, maturation, and function of various tissues throughout the body, including the peripheral and central nervous systems [131]. These hormones have been found to possess significant neuroprotective properties in various neurodegenerative conditions [132–135]. Estrogen treatment [133,136–141] was found effective in decreasing neurological sequel following MCAO and reduced the incidence of Alzheimer’s disease in postmenopausal women [133,142]. Progesterone has been proposed to possess neuroprotective properties in both ischemic stroke [138] and TBI in rats [143–147]. Despite the prevailing evidence of estrogen and progesterone acting as a neuroprotective agent, the mechanism by which estrogens exert their neuroprotective actions is unknown, with a plethora of cellular and extracellular actions being suggested.

Estrogen and progesterone may at least partially, mediate their neuroprotective properties via a glutamate-related mechanism. Several recent findings support this hypothesis. In human volunteers, of all factors examined (age, time elapsed from the last meal or drink, gender, daily physical activity, recent coffee consumption *etc*.) only female gender was associated with significantly lower blood glutamate levels [47]. Stover and Kempski found that following isoflurane anesthesia in neurosurgical procedures, glutamate concentrations were more prominently elevated in male patients than in females [54]. Furthermore, it has been shown that female patients suffering from ALS [16,85] have significantly lower plasma glutamate concentrations than males. Estrogen and progesterone have been found to inversely correlate with plasma glutamate concentrations along the course of the female menstrual cycle [48]. As the menstrual cycle begins, plasma estrogen and progesterone levels are very low [148,149] with relatively elevated glutamate (above baseline), which still remains lower than in males. Later during the cycle, blood glutamate decreases, coinciding with rising plasma estrogen and progesterone levels. At the hormonal peak, blood glutamate levels drop to almost half the initial values. In another study, pregnancy related elevation of female hormones led to blood glutamate levels during the 2nd and 3rd trimesters that were significantly lower than in the 1st trimester [48]. A recent study in a rat model of TBI demonstrated the effect of estrogen treatment and pretreatment on glutamate levels and outcomes in male rats injected with estrogens, which lead to significant and sustained decrease of blood glutamate levels in all the groups, correlating with improved neurological outcomes [150].

### 4.5. Extracorporeal Methods of Glutamate Elimination

Extracorporeal methods of glutamate elimination (hemodialysis, hemofiltration and peritoneal dialysis) have several important advantages. First and foremost, these methods definitively eliminate, rather than redistribute or reversibly converse, excess glutamate from different compartments. Second, considering that the majority of pharmacological options listed above cannot be used for treatment in humans, since those options have not yet undergone the required safety trials, extracorporeal methods that have been widely used in many conditions in ICU patients may therefore be applied for this indication. Extracorporeal removing systems, such as hemodialysis effectively remove many amino acids including glutamate, improving the amino acid profile towards the norm [69–71,151]. Similar extracorporeal removing systems may be usefully applied precisely for elimination of glutamate excess in different neurodegenerative conditions and to provide neuroprotection. Unfortunately, hemodialysis may have several limitations: Firstly, a significant proportion of the patients who are admitted suffer from hemodynamic instability, particularly hypovolemia, or are in shock. Secondly, anticoagulation, which may be detrimental for patients suffering from multiple trauma or isolated head injury because of the risk of bleeding, is preferred for hemodialysis procedures in order to prevent clot formation in the set’s tubing. Continuous hemofiltration may circumvent some of these limitations.

### 4.6. Hypothermia

Hypothermia is a controversial treatment modality in different neurological disorders including TBI, stroke and global ischemia [152–160], which may or may not be associated with improved neurological outcomes. Several mechanisms may be involved in hypothermia induced neuroprotection including: decreased cerebral metabolic rate, decreased cerebral blood flow and lower intracranial pressure, limited free oxygen radical formation, and decreased glutamate release in brain [161]. Recently we examined the effect of moderate hypothermia (30°–32° Celsius) on blood glutamate levels in naive rats. This led to a 40% reduction of glutamate, and lasted up to 9 h. This process was accompanied by elevation of plasma GOT and GPT levels which may contribute to the decrease of blood glutamate levels via facilitated conversion of glutamate into 2-ketoglutarate by those enzymes. Previous cold exposure was shown to lead to multifold elevation of plasma GOT and GPT activity in rats [118]. Overall, a plethora of methods utilizing different mechanisms have been shown to decrease blood and ECF glutamate concentrations in experimental settings. Thus, decreased blood glutamate concentration may be associated with improved neurological outcomes irrespective of the precise process, which was involved in reducing glutamate concentration. Although some may be promising as treatment modalities, human clinical trials yet need to be performed in order to establish glutamate scavenging as an acceptable treatment.

## 5. Factors Elevating Blood Glutamate Levels

Elevated blood glutamate levels may interfere with normal brain-to-blood glutamate efflux and may therefore worsen neurological outcome under different neurological conditions [59–61,162]. Thus, factors elevating blood glutamate levels should be avoided in situations where glutamate has been shown to exhibit its neurotoxic properties. Meals rich in monosodium glutamate have been shown to elevate circulating blood and muscle glutamate levels in both humans and animals [128,163]. Interestingly, ingested glutamate increases plasma but not RBC concentration, since glutamate is not transferred into RBC, but is rather formed in the RBC from glutamine by glutaminase [112]. Ordinary meals, not enriched with monosodium glutamate, do not significantly influence blood or plasma glutamate levels due to a very fast first pass metabolism in gut and liver [128]. An elevated aspartate concentration in this study reflected enhanced breakdown of glutamate. Physical exercise may be a powerful stimulus to elevate glutamate concentration in the blood in both animals [164] and humans. Studies in athletes and laymen submitted to extreme physical exercise found a pattern of initial elevation of glutamate and alanine, followed by a decrease in blood levels throughout the recovery process [165–168]. Glutamate concentration in skeletal muscle has been shown to be 100 times higher than in plasma [72]. This may be of importance in clinical scenarios such as in cases of multi-trauma, crush injuries, compartment syndrome, severe hyperthermia, heat stroke and rhabdomyolisis where extensive muscle damage may accompany brain injury.

Severe hyperthermia was associated with a multifold elevation of blood glutamate in rats submitted to severe hyperthermia by external heating. We postulated that this elevation was a heat-mediated sequence of muscle injury [169]. This idea was supported by a significant elevation of markers of muscular damage, myoglobin and CPK. In the circumstance of uncontrolled massive shifting of glutamate from damaged muscle, blood glutamate scavengers, oxaloacetate and pyruvate were ineffective in decreasing blood glutamate levels. It cannot be ruled out that a worse neurological outcome associated with hyperthermia after brain insults may be at least partially determined by elevation of glutamate [169]. Long lasting immobilization stress in rats has been shown to elevate their plasma glutamate levels [170].

Several hormones have been associated with elevation of plasma glutamate levels. Both hydrocortisone [62,171] and thyroid [62,171] hormones were shown to elevate plasma glutamate concentration in rats. In humans, hyperthyroidism also raises blood glutamate levels, but hypothyroidism has no blood glutamate reducing effect [171]. In patients with growth hormone deficiency, blood glutamate levels were increased [172].

## 6. Neuroprotective Role of the Placenta

Birth asphyxia is a leading cause of neurological pathology and death in newborns [173]. Elevated glutamate concentrations in the fetal brain’s extracellular fluids (ECF) are associated with a worse neurological outcome after fetal asphyxia [173–175]. The placenta has an abundance of glutamate transporters and is capable of removing an excess of glutamate by active transport from the fetal to maternal blood [176,177]. Amino acid concentrations in the fetus are higher compared with maternal concentrations [177,178]. Glycine is transported from the mother into the fetal circulation by active transport and is converted to glutamate predominantly in the fetal liver. When excess glutamate is present in the fetal circulation, the glutamate is transported to the placenta, which is responsible for the metabolism of as much as 80 percent of fetal glutamate [176,177], thus preventing glutamate-induced neurotoxicity. The glutamate transporters EAAT1, EAAT 2, and EAAT 3 located in the placenta, are key components of the glutamate-glutamine cycle responsible for glutamate transport across cell membranes [176]. Considering that the placenta has a well-developed system of transporters for glutamate elimination, it seems plausible that one may create a favorable chain of glutamate concentration gradients that would ultimately lead to the elimination of excess glutamate from the fetal brain. This chain would start with the reduction of glutamate concentrations in the maternal blood compartment, thereby creating a favorable glutamate gradient between the maternal and fetal blood. This would lead to a placenta-mediated efflux of glutamate from the fetal to maternal blood. Decreases in fetal blood glutamate concentrations should then facilitate the efflux of excess of glutamate from the fetal brain’s ECF compartment, thereby preventing its neurotoxic effects. Such an approach may offer new therapeutic insights into fetal blood glutamate reduction *in utero* by way of reducing maternal glutamate levels.

## 7. Conclusion

Elevated brain interstitial glutamate is associated with a wide variety of brain insults, while the reduction of excess glutamate has been shown to minimize secondary injury and improve neurological outcomes. Eliminating excess glutamate from the brain by increasing its removal into the blood has been a target of numerous recent studies. Scavenging glutamate from the blood, irrespective of the means, increases the blood-to-brain glutamate gradient, thus enhancing its removal from brain to blood. Experimental models have shown that utilizing this pathway is beneficial and improves neurological outcomes. Further studies investigating these pathways are needed in order to clarify the specific mechanisms involved and to pave the way towards human clinical studies and, further on, to practical use in treatment.

## Supplementary Materials



## Abbreviation

CRF: Chronic Renal Failure
IGFBP: Insulin Like Growth Factor Protein
TBI: Traumatic Brain Injury
MCAO: Middle Cerebral Artery Occlusion
CT: Computed Tomography
MRI: Magnetic Resonance Imaging
IGF: Insulin like Growth Factor
RBC: Red Blood Cells
HIV: Human Deficiency Virus
ALS: Amytrophic Lateral Sclerosis
ARF: Acute Renal Failure
CRF: Chronic Renal Failure
IGFBP: Insulin Like Growth Factor Protein
TBI: Traumatic Brain Injury
MCAO: Middle Cerebral Artery Occlusion
CT: Computed Tomography
MRI: Magnetic Resonance Imaging
IGF: Insulin like Growth Factor
RBC: Red Blood Cells
HIV: Human Deficiency Virus
ALS: Amytrophic Lateral Sclerosis
ARF: Acute Renal Failure
