# Supplementary material for: Blood Glutamate Scavenging: Insight into Neuroprotection
Source: Int J Mol Sci. 2012 Aug 13;13(8):10041–66. doi: 10.3390/ijms130810041 (PMC3431845; doi:10.3390/ijms130810041)
Supplement: Supplementary file 1 [file ijms-13-10041-s001.pdf]

Blood Glutamate Scavenging: Insight into Neuroprotection

Supplementary Information

Figure S1. Homeostasis of glutamate in brain extracellular fluids: glutamate is maintained at around 1 μM in brain interstitial and CSF in spite of the 10^4 to 10^5 times larger concentration in brain cells (~10 mM) and synaptic vesicles (~100 mM). This is due to the presence of Na^+-dependent EAATs not only on neuronal and glial cells but also on the brain vasculature. There is evidence for the presence of EAATs at the astroglial end feet facing both the neuropil and the abluminal membrane of capillary endothelial cells. The end feet take up from brain ISF excess glutamate which is transformed into Gln via the Gln synthetase (1). Gln exits from the end foot through system N transporters and is taken up by neutral amino acid transporters (2) present at the abluminal membrane of capillary endothelial cells. Gln is transformed by a glutaminase (3) into glutamate which diffuses through facilitative transport (4) into blood. Although not shown in the intact brain *in situ*, the endothelial cells may harbor EAATs (5) that could under specific physiological and pathological conditions, directly transport glutamate into blood. In such an event, the glutamate transport in endothelial cells could be concentrative and of high affinity. The antiluminal transporters would thus take up glutamate, concentrate it to a concentration well above the glutamate plasma levels (40 μM) allowing glutamate to flow into blood by facilitated transport down a concentration gradient. Glutamate scavengers, by distinct mechanisms, decrease plasma concentration of glutamate, thus enhancing this concentration gradient, removing excess deleterious glutamate from brain ECF and CSF. Copyright (2009), Elsevier [1].

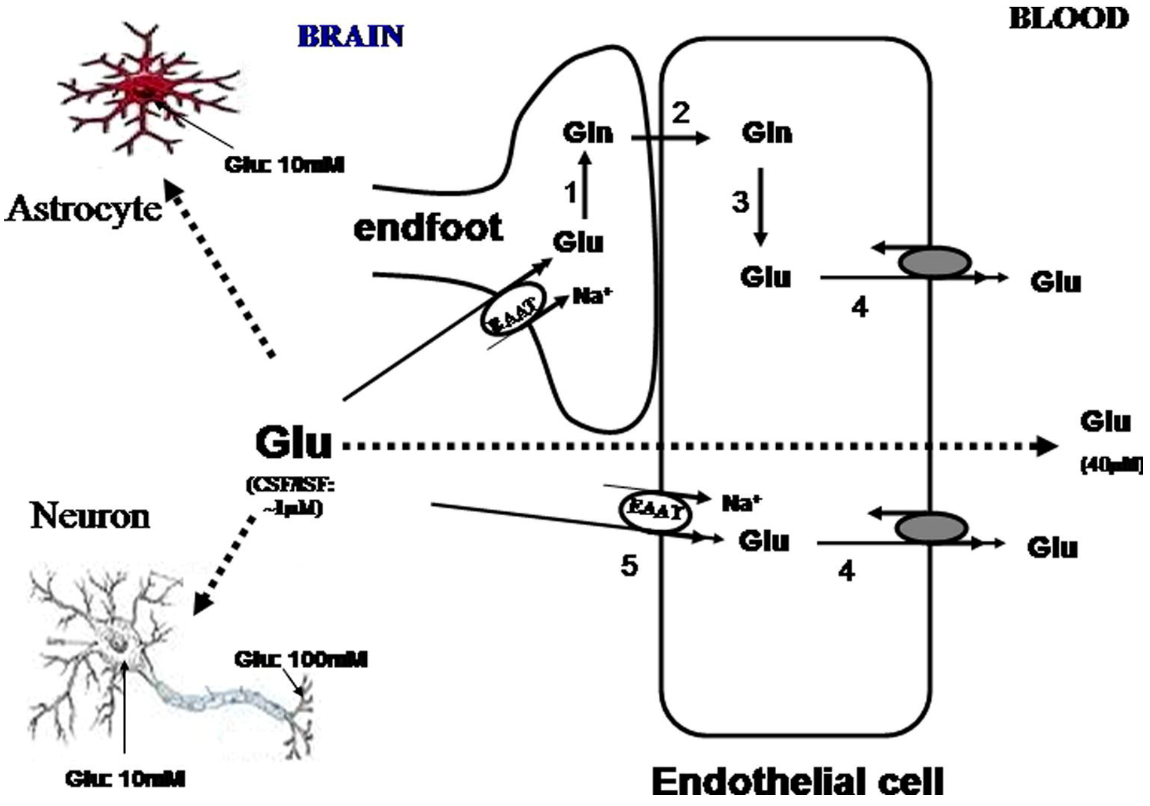

Reference

1. Teichberg, V.I.; Cohen-Kashi-Malina, K.; Cooper, I.; Zlotnik, A. Homeostasis of glutamate in brain fluids: An accelerated brain-to-blood efflux of excess glutamate is produced by blood glutamate scavenging and offers protection from neuropathologies. *Neuroscience* **2009**, *158*, 301–308.

© 2012 by the authors; licensee MDPI, Basel, Switzerland. This article is an open access article distributed under the terms and conditions of the Creative Commons Attribution license (http://creativecommons.org/licenses/by/3.0/).
